# A Case of Complicated Bacteremia: When the Source Is in the Blood

**DOI:** 10.7759/cureus.79782

**Published:** 2025-02-27

**Authors:** Raquel Costeira, Elisa Macedo Brás, Ricardo Manuel Pereira, Inês Barbosa Leão, Catia Canelas

**Affiliations:** 1 Internal Medicine, Unidade Local de Saúde de Trás-os-Montes e Alto Douro, Vila Real, PRT

**Keywords:** aortic stenosis, bacteremia, bacterial translocation, cirrhosis, endocarditis, enterococcus faecium

## Abstract

Infective endocarditis is an infectious disease of the heart tissue, mainly affecting heart valves and intracardiac devices. We present the case of a 71-year-old male pacemaker carrier with a history of hepatic cirrhosis, esophageal varices, hepatocellular carcinoma, and severe aortic stenosis, who was admitted to the emergency room and hospitalized due to upper gastrointestinal bleeding. Although upper endoscopy showed no signs of active acute hemorrhage, the patient required a red blood cell transfusion. Upon admission, elevated inflammatory parameters prompted the initiation of empirical therapy with ceftriaxone. Although urinalysis, chest X-ray, thoracoabdominopelvic computed tomography, and transthoracic echocardiogram weren't suggestive of infection, an *Enterococcus faecium* was isolated in blood cultures. Following an antibiotic switch to daptomycin, based on susceptibility testing, and the patient's hemodynamic stability, he was transferred to a home hospitalization unit for continued care. Despite good clinical and analytical progress, the patient's history of aortic valve stenosis and pacemaker, along with persistently positive blood cultures despite antibiotic therapy and sustained fever, raised a high level of clinical suspicion. This led to the decision to perform a new echocardiogram, which revealed several aortic valve vegetations, allowing the diagnosis of infective endocarditis. Following a multidisciplinary discussion, and in accordance with antibiotic susceptibility tests, linezolid was initiated. After 40 days, although the echocardiogram was still suggestive of aortic valve infiltration due to an infectious process, hemodynamic stability, sustained apyrexia, and sterile blood cultures allowed for a possible discharge on oral therapy with moxifloxacin and rifampicin. Although this is a case of infective endocarditis in a high-risk patient, the chronology is unusual, as endocarditis was not detected in the initial echocardiogram. The diagnosis was only possible after weeks of persistent positive blood cultures, thanks to the medical team's high level of suspicion, which led them to insist on repeating the echocardiogram. In fact, the diagnosis of infective endocarditis remains a challenge to this day. This case highlights the importance of recognizing risk factors and pursuing the diagnosis when clinical suspicion persists, including repeating imaging when necessary to ensure timely diagnosis and appropriate management.

## Introduction

Infective endocarditis (IE) is an infectious disease affecting the heart tissue, primarily targeting heart valves and intracardiac devices [1]. It represents both a diagnostic and a public health challenge, with an incidence of 13.8 cases per 100,000 individuals per year in 2019 and 66,300 deaths worldwide [2]. Its incidence has progressively increased over the past decades. Currently, it affects most often men and older patients (over 65 years old) [3].

Predisposition for this infection usually results from a combination of certain conditions, including the presence of a structure that can be colonized by bacteria, the existence of pathogens in the bloodstream, and a host immune response that facilitates its development. The risk of IE development is even greater in patients who have a previous history of the disease. Various bacterial entry points have been described, including infections of the skin, oral cavity, and gastrointestinal or genitourinary system, intravenous drug use, unprotected vascular puncture, indwelling cardiac device or intravenous catheter, and other invasive diagnostic and therapeutic procedures performed in healthcare facilities [1].

Diverse are the bacteria usually responsible for an IE. In Europe,streptococci are responsible for 31% of cases, followed by *Staphylococcus aureus* (28%), coagulase-negative staphylococci (13%), and cases of culture-negative endocarditis (10%) [3]. Other important bacteria, although less frequent in Europe, include *Enterococcus*, especially in cases of native valve infection, accounting for 11% of cases worldwide [4]. These include *Enterococcus faecium* bacteremia, primarily resulting from gastrointestinal infections or catheters, which appears to have a low association rate with IE (5% of cases) [5].

In addition to presenting a rare case of IE caused by *Enterococcus faecium*, this case highlights the diagnostic challenges of IE, particularly in a complex patient with a history of aortic stenosis and cirrhosis, when initial imaging is negative.

## Case presentation

We present a case of a 71-year-old male patient with a history of severe aortic stenosis (left ventricular ejection fraction of 56%, aortic valve area of 0.9 cm^2^, mean gradient of 39 mmHg, and peak aortic velocity of 4 m/s), essential arterial hypertension, and third-degree atrioventricular block, which required a pacemaker implantation a year prior. He also had type 2 diabetes mellitus. Other relevant medical history included cirrhosis with a Child-Pugh score of A6 and Model for End-Stage Liver Disease (MELD)-Na score of 10, due to MetALD (overlap of metabolic dysfunction-associated steatotic liver disease (MASLD) and alcohol-related liver disease (AALD)). The patient had clinically significant portal hypertension and a history of esophageal variceal bleeding and was further complicated with Barcelona Clinic Liver Cancer (BCLC) stage C hepatocellular carcinoma and portal and mesenteric vein thrombosis, with patients usually medicated with metformin/sitagliptin, spironolactone, carvedilol, and enoxaparin. 

This patient presented to the emergency room (ER) with new-onset melena, without any other symptoms. He denied fever, shivering, malaise, headache, myalgias, arthralgias, night sweats, or dyspnea. On physical examination, he appeared pale, with a blood pressure of 95/51 mmHg. There was no tachycardia or signs of abdominal peritoneal irritation. Pulmonary and cardiac auscultation were physiological, with no heart murmurs. 

Blood tests (Table 1) revealed a normochromic normocytic anemia with thrombocytopenia and neutrophilia (8.65×10^3^/uL neutrophils), a normal procalcitonin value, and a slightly increased C-reactive protein (2.3 mg/dL). Serum chemistries exhibited hyperglycemia (586 mg/dL) with hyperlactatemia (11), but no acidemia. Liver function tests were within normal limits, with a cholestatic pattern of change in liver biochemistry values. Renal function tests presented a high urea value (181 mg/dL) with normal serum creatinine of 0.9 mg/dL, probably related to gastrointestinal bleeding. Additionally, hyperkalemia of 6.7 mEq/L and hyponatremia of 126 mEq/L were noted. 

**Table 1:** Hemogram and serum chemistries obtained in the ER, on admission ER: emergency room; AST: aspartate aminotransferase; ALT: alanine aminotransferase; GGT: gamma-glutamyl transferase; CK: creatine kinase; pro-BNP: pro-B-type natriuretic peptide; INR: international normalized ratio

Parameter	Value	Reference value
Hemoglobin (g/dL)	9.2	13-18 g/dL
Leukocytes (×10^3^/µL)	10.15	4-11×10^3^/µL
Platelets (×10^3^/µL)	136	150-400×10^3^/µL
Glucose (mg/dL)	586	82-115 mg/dL
Albumin (g/dL)	2.7	3.4-4.8 g/dL
Potassium (mEq/L)	6.7	3.7-5.1 mEq/L
Sodium (mEq/L)	126	135-147 mEq/L
Chlorine (mEq/L)	87	96-106 mEq/L
Urea/creatinine (mg/dL)	181/0.9	<50/0.7-1.4 mg/dL
AST/ALT (U/L)	43/46	<40/<41 U/L
GGT (U/L)	112	10-49 U/L
Alkaline phosphatase (U/L)	169	40-130 U/L
Total bilirubin (mg/dL)	1.00	<1.2 mg/dL
Direct bilirubin	0.4	<0.3 mg/dL
C-reactive protein (mg/dL)	2.3	<0.5 mg/dL
Procalcitonin (ng/mL)	0.8	<0.5 ng/mL
Troponin T (ng/mL)	0.03	<0.05 ng/mL
Myoglobin (ng/mL)	48.7	<72 ng/mL
CK-MB (ng/mL)	3.12	<4.9 ng/mL
Pro-BNP (pg/mL)	1284	<120 pg/mL
INR	1.12	<1.2

Due to the high probability of gastrointestinal bleeding with hemodynamic repercussion, fluid resuscitation was initiated, along with 2 g of ceftriaxone, 40 mg of intravenous pantoprazole, and 200 mg of thiamin. Besides, insulin therapy (a total of 20 units) was initiated to correct hyperglycemia. An upper endoscopy was performed, revealing esophageal scars from previous ligation, with no signs of bleeding or active hemorrhage. The stomach contained abundant gastric stasis, obstructing visualization and preventing further examination of this segment.

With the diagnosis of acute decompensation of hepatic cirrhosis with acute gastrointestinal bleeding, potentiated by therapeutic hypocoagulation for prior portal and mesenteric thrombosis, the patient was referred to the intensive care unit (ICU). Due to his fragile clinical condition, advanced liver disease complicated by neoplasia, and low functional reserve to withstand more invasive interventions, ICU admission was considered inappropriate. Following the stabilization of the patient's blood pressure and no further episodes of bleeding, the patient was hospitalized in the Internal Medicine department. 

During the hospital stay, a drop in the hemoglobin value to 7.2 g/dL with hemodynamic instability led to an acute blood transfusion necessity, with the transfusion of two units of packed red blood cells. No new hemorrhage episodes were observed. Due to the absence of hematochezia and a recent normal colonoscopy result, it was determined, following a multidisciplinary discussion with the Gastroenterology team, that there was no indication to repeat the exam.

From the septic screening performed upon admission, before the onset of fever spikes, three pairs of blood cultures tested positive for *Enterococcus faecium* bacteremia. The strain was sensitive only to daptomycin, quinupristin/dalfopristin, teicoplanin, vancomycin, and linezolid (Table 2) while resistant to penicillin, ampicillin, ciprofloxacin, and levofloxacin. Although a clear source for bacteremia was not identified, ceftriaxone was switched to intravenous daptomycin (500 mg, twice daily). 

**Table 2:** Antibiotic sensitivity testing of Enterococcus faecium isolated from the patient's blood cultures MIC: minimum inhibitory concentration

*Enterococcus faecium* sensitivity	
MIC≤1	Vancomycin
	Quinupristin/dalfopristin
MIC≤2	Teicoplanin
	Linezolid
MIC=2	Daptomycin

Searching for an infectious origin, other studies were performed. Chest X-ray was clear, and urine culture was also negative. A thoracoabdominopelvic CT scan was executed, but no evidence of an infectious process was found. No signs of IE were also found in the transthoracic echocardiogram (TTE) performed. 

**Figure 1:**
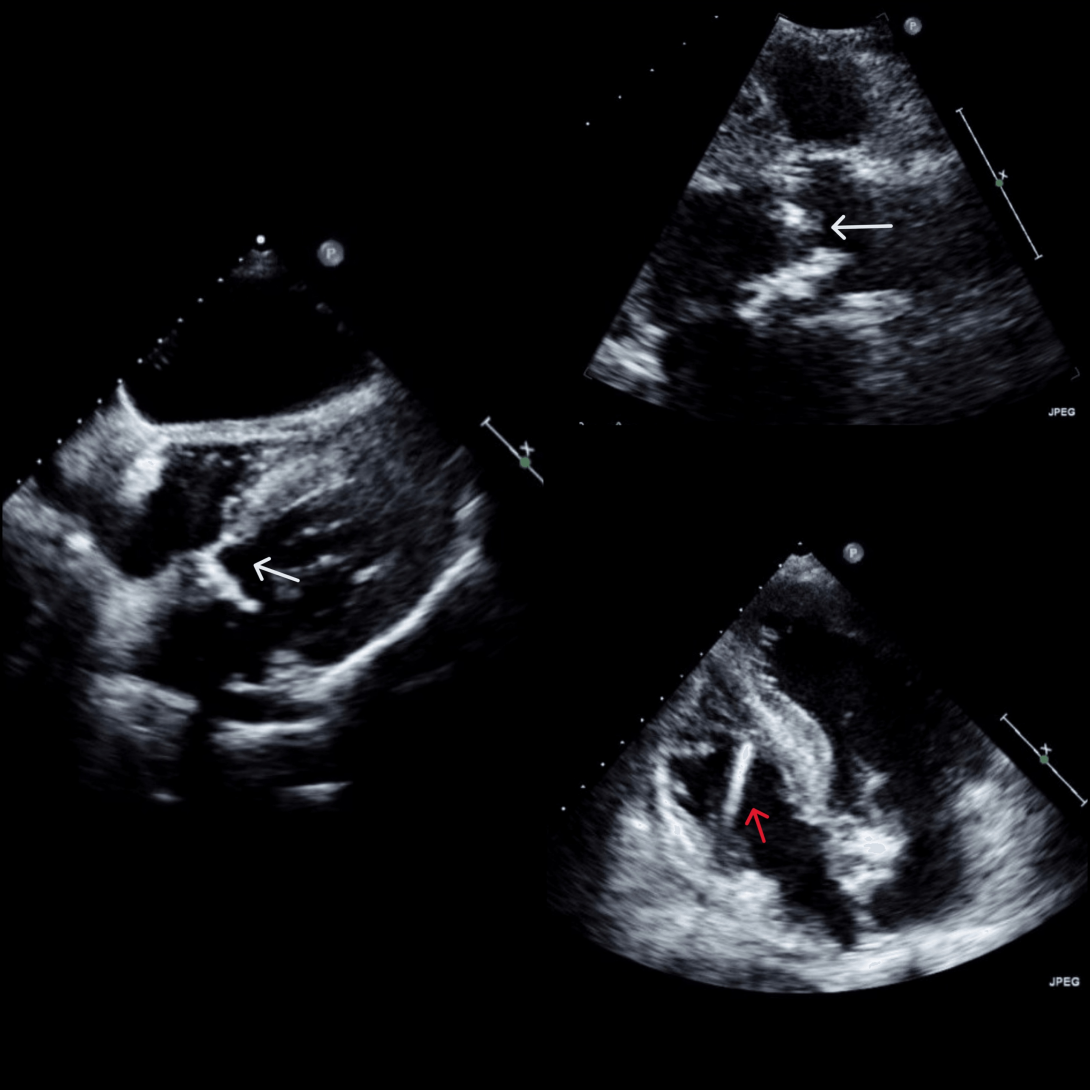
TTE showing aortic valve vegetations as mobile echogenic structures (white arrows), visible in the parasternal long-axis view (left and upper right images). Additionally, the pacemaker wire (red arrow) is seen in the right heart chambers without vegetations, as observed in the apical four-chamber view (bottom right image) TTE: transthoracic echocardiogram

Following clinical and analytical improvement, the patient was transferred to a home hospitalization unit (HHU). Despite the antibiotic therapy switch and three weeks of adjusted antibiotic, the C-reactive protein remained elevated (8.6 mg/dL), and blood cultures persistently tested positive for the same bacteria. The patient was febrile, with no other symptoms or signals found. The Modified Duke Criteria was calculated, with a score of 1 major (isolation of a microorganism that occasionally or rarely causes IE from three or more separate blood culture sets) and 2 minor criteria (predisposition and fever), with possible IE diagnosis. Although TTE sensitivity can be relatively low, due to the hemorrhagic risks associated with performing a transesophageal echocardiogram in this patient, given the recent gastrointestinal bleeding, the medical team initially opted to repeat a TTE instead. This revealed, for the first time, several filiform structures suggestive of vegetations, one being approximately 0.5 cm in diameter, with doubt of a possible image of a periaortic abscess (Figure 1). The presence of flow in the most anterior area of ​​the leaflets leading to mild regurgitation admitted possible perforation. With the TTE results, a major criterion (imaging evidence of vegetation on echocardiography) was added to the Modified Duke Criteria, allowing for the diagnosis of definite endocarditis. 

Given the patient's frailty and multiple comorbidities, including advanced liver disease complicated by hepatocellular carcinoma and a diagnosis of IE, a multidisciplinary team comprising Internal Medicine, Cardiology, and Cardiothoracic Surgery decided against surgical intervention, as the risks of the procedure outweighed the potential benefits. Instead, a conservative approach with six weeks of targeted antibiotic therapy was chosen.

As the patient was not responding to daptomycin and to improve antibiotic penetration into the vegetation, therapy was switched to intravenous linezolid (600 mg, twice daily). After 40 days, follow-up blood cultures returned negative for the first time. Although echocardiographic findings remained positive for IE, the patient showed signs of improvement, including sustained apyrexia, a decrease and normalization of inflammatory markers, hemodynamic stability, and sterile blood cultures. Due to probable linezolid-induced pancytopenia and based on the Partial Oral Treatment of Endocarditis (POET) study, which demonstrated that in stable patients with left-sided endocarditis, switching to oral antibiotic therapy was non-inferior to continued intravenous treatment, therapy was transitioned to moxifloxacin (400 mg, once daily) and rifampicin (600 mg, twice daily). [6] This allowed for a safe discharge. 

The patient was re-evaluated after two weeks of discharge in internal medicine consultation and palliative care, showing signs of clinical improvement.

## Discussion

In the case presented, the initial presentation was not strongly indicative of IE. Predisposing factors for IE typically include pre-existing valvular or congenital heart disease and surgically or transcatheter implanted prosthetic valves, especially bioprostheses and ventricular assist devices [1]. This case highlights the challenges of diagnosing and managing IE in complex patients, particularly those with cirrhosis, a history of aortic stenosis, and a pacemaker. In this case, both severe aortic stenosis and the presence of a pacemaker were significant risk factors for IE. Although blood cultures were positive since admission, the first TTE didn't confirm signs of valvular vegetations or signs of pacemaker lead infection. Apart from fever, the clinical examination revealed no signs of embolization or heart murmurs suggestive of IE nor any indications of more common sources of bacteremia, such as catheter-related infection. Additionally, validated scoring systems only suggested possible IE diagnosis. On the other hand, the agent isolated in blood cultures was not a frequent causative agent of IE. That said, the diagnosis was only possible because clinical suspicion remained high even with these results, essentially driven by the persistent positive blood cultures, despite targeted antibiotic therapy, showing an inadequate source control. Although this presents a case of IE in a high-risk patient, the chronology presented is rare, as it was just in the second TTE that IE was observed, even with persistently positive blood cultures before. One possibility for this happening is IE not being, in fact, present in the first evaluation. Although the source of bacteremia was not obvious in this case, it is important to remember that infections are common and potentially severe complications of end-stage liver disease, including bloodstream infections [7]. Physiologically, low-grade bacterial translocation can occur and is usually neutralized by gut-associated lymphoid tissue, systemic immunity, or the liver [8]. However, in cirrhotic patients, bacterial overgrowth due to portal hypertension and intestinal hypomotility can lead to dysregulated bacterial translocation from the gastrointestinal tract. This process is facilitated by increased intestinal mucosal permeability, impaired hepatic filtration of bacteria, and immune dysfunction [9]. In the case presented, a possible source of bacteremia presented on admission was hematogenous dissemination via the gastrointestinal tract, which, being persistently positive after weeks, led to an IE development. Another possibility is that IE was not detected at first. In fact, the sensitivity of TTE can be relatively low (approximately 55% for native valves and lower for prosthetic valves). Difficulties exist with the differentiation of small vegetations from degenerative valve disease, which can lead to inconclusive TTE and the need for a transesophageal echocardiography (TEE), as it has a higher sensitivity and specificity, allowing a diagnosis exclusion [4]. In the presented case, the high risk of hemorrhagic complications associated with TEE led to the decision to repeat TTE instead of performing an earlier TEE. Ultimately, the TTE revealed imaging findings consistent with IE.

Enterococci are gram-positive, facultatively anaerobic bacteria and represent one of the most important leading causative microorganisms of bloodstream infections, especially in male, older, fragile patients, with renal impairment, liver disease, and solid organ and bone marrow transplant [10]. This group includes* Enterococcus faecalis* and *Enterococcus faecium*, natural inhabitants of the gastrointestinal, hepatobiliary, and genitourinary tracts. Of both, *Enterococcus faecalis* is most frequently associated with IE [11]. In fact, cases of IE with isolates of *Enterococcus faecium *mainly include cases of vancomycin-resistant enterococci and nosocomial infections [12]. In addition,* Enterococcus faecium* is also linked to patients with more severe illness, has higher rates of antibiotic resistance, and has been associated with higher mortality than *Enterococcus faecalis*, being more frequent in liver cirrhotic patients or patients with previous broad-spectrum antibiotic treatment, with origin in gastrointestinal infections or catheters [10]. There is very little evidence concerning *Enterococcus faecium* bacteremia, but it appears to have a low association rate with IE. In fact, the 2023 Duke-International Society for Cardiovascular Infectious Diseases (ISCVID) Criteria [12] presented a revised Duke Criteria, not considering anymore *Enterococcus faecium* as a "typical endocarditis pathogen", being nowadays two positive blood cultures for this pathogen considered as a minor criterion [13]. In terms of treatment, linezolid and daptomycin are viable options for strains resistant to penicillin and vancomycin [3]. In our case, despite the absence of vancomycin resistance or a nosocomial setting, the patient's comorbidities, particularly cirrhosis, made *Enterococcus faecium *a plausible pathogen. When IE was first considered as a possible diagnosis, the patient met a major criterion by having three or more separate blood culture sets positive for the pathogen, even though a typical IE-causing agent was not isolated. However, had the required number of positive blood cultures not been met, the patient would not have fulfilled the criteria for possible endocarditis. Even with vegetation detected on TTE, the Duke Criteria would have only supported a possible, rather than a definite, IE diagnosis. This case underscores the crucial role of blood cultures and the importance of maintaining a high level of clinical suspicion, particularly in patients who meet major imaging criteria and present with significant bacteremia, regardless of the bacterial species.

Lastly, this case highlights the importance of tailoring therapy to each individual patient. In the treatment of IE, a careful balance must be struck between the risks and benefits of different management strategies, including antibiotic selection, route of administration, treatment duration, and surgical eligibility. In clinical practice, many patients who meet guideline-based indications for surgery do not ultimately undergo the procedure. As seen in this case, despite a Child-Pugh score of A6 and a MELD-Na score of 10, the presence of advanced liver disease complicated by hepatocellular carcinoma, portal vein thrombosis, and gastrointestinal bleeding, along with the patient's other comorbidities, made the surgical risk unacceptably high. Given these factors, a more conservative approach was considered the most appropriate course of action.

Even with antibiotic therapy, the risks associated with prolonged treatment and hospitalization are significant. The HHU played a crucial role in this case, enabling the observation, diagnosis, and management of a fragile patient. HHUs are increasingly emerging worldwide as an alternative to inpatient hospital care, aiming to reduce hospitalization time. In the case presented, the patient met the geographic criteria, residing within the HHU catchment area; the social criteria, being in a nursing home facility; and the clinical criteria, as the patient was clinically stable at the time of transfer. However, the development of sustained fever and the subsequent diagnosis of IE posed a significant challenge within the HHU setting. The patient's residence in a nursing home with access to a skilled team of internists played a crucial role in successfully managing this challenge within the HHU framework, enabling the earlier recognition and prompt management of complications.

## Conclusions

Despite well-established diagnostic criteria and its high morbidity and mortality, IE remains a diagnostic challenge, with blood cultures as the cornerstone of diagnosis. Although *Enterococcus faecium* is a less common IE pathogen and was excluded from the revised 2023 Duke-ISCVID Criteria, clinicians should remain vigilant in at-risk patients, especially in cases of persistent bacteremia. Critical thinking and awareness of key risk factors are essential for timely diagnosis and appropriate imaging selection while avoiding unnecessary echocardiographic evaluations. Whenever possible, treatment decisions should be guided by clinical evidence, along with a thorough understanding of bacterial virulence and infection pathogenesis.
